# Ten-Year Survival Rate of 82% in 262 Cases of Arthroscopic Bone Marrow Stimulation for Osteochondral Lesions of the Talus

**DOI:** 10.2106/JBJS.23.01186

**Published:** 2024-05-10

**Authors:** Quinten G.H. Rikken, Margot B. Aalders, Jari Dahmen, Inger N. Sierevelt, Sjoerd A.S. Stufkens, Gino M.M.J. Kerkhoffs

**Affiliations:** 1 Department of Orthopedic Surgery and Sports Medicine, Amsterdam UMC, University of Amsterdam, Amsterdam, The Netherlands; 2 Sports and Musculoskeletal Health Programs, Amsterdam Movement Sciences, Amsterdam, The Netherlands; 3 Academic Center for Evidence-based Sports Medicine (ACES), Amsterdam UMC, Amsterdam, The Netherlands; 4 Amsterdam Collaboration for Health and Safety in Sports (ACHSS), International Olympic Committee (IOC) Research Centre, Amsterdam UMC, Amsterdam, The Netherlands; 5 Orthopedic Department, Spaarne Gasthuis Academy, Hoofddorp, The Netherlands

## Abstract

**Background:**

The long-term sustainability of arthroscopic bone marrow stimulation (BMS) for osteochondral lesions of the talus (OLT) remains a matter of debate. The primary aim of the present study was to assess the 10-year survival free from revision in ankles that had undergone arthroscopic BMS for an OLT. The secondary aim was to evaluate the influence of baseline patient and lesion characteristics on survival.

**Methods:**

Patients who underwent arthroscopic BMS for a symptomatic OLT and had a minimum follow-up of 10 years were included to assess procedure survival. The primary outcome, the 10-year cumulative survival rate, was analyzed by the Kaplan-Meier survival method. Secondary outcomes were the median time to revision and the effects of baseline factors (lesion size, primary or non-primary lesion type, preoperative cysts, and obesity as defined by a body mass index [BMI] of ≥30 kg/m^2^) on survival, analyzed with a Cox regression model and reported using hazard ratios (HRs).

**Results:**

The 262 included patients had a mean follow-up of 15.3 ± 4.8 years. The 10-year cumulative survival rate of the arthroscopic BMS procedures was 82% (95% confidence interval [CI]: 77% to 87%). At 15 years of follow-up, the cumulative survival rate was 82% (95% CI: 76% to 86%). The median time to revision was 2.4 years (interquartile range: 1.3 to 5.1 years). Of the baseline factors, obesity (HR: 3.0 [95% CI: 1.44 to 6.43], p < 0.01) was associated with decreased survival. Lesion size (HR: 0.9 [95% CI: 0.5 to 1.8], p = 0.8), non-primary lesion type (HR: 1.8 [95% CI: 0.9 to 3.4], p = 0.1), and the presence of preoperative cysts (HR: 1.0 [95% CI: 0.6 to 1.9], p = 0.9) were not significantly associated with survival.

**Conclusions:**

At a minimum follow-up of 10 years, the survival rate of arthroscopic BMS for OLT was 82%. At 15 and 20 years of follow-up, survival appeared to remain stable. Obesity (BMI ≥ 30 kg/m^2^) was associated with a higher likelihood of revision surgery. This risk factor should be incorporated into the treatment algorithm for OLT when counseling patients regarding surgery.

**Level of Evidence:**

Therapeutic Level IV. See Instructions for Authors for a complete description of levels of evidence.

Osteochondral lesions of the talus (OLT) are lesions of the articular cartilage and the underlying subchondral bone. Symptomatic OLT typically result in pain and can be debilitating for patients, especially for those involved in physical activities and sports. Up to 75% of OLT are the sequelae of trauma, such as a sprain or fracture, and the lesions may initiate the cascade toward end-stage ankle osteoarthritis^1-3^.

Nonoperative treatment is the first-line treatment for OLT, but it fails in up to 55% of patients, meaning that the majority of patients require surgical treatment^4,5^. In smaller (<150 mm^2^) primary lesions, the preferred surgical treatment for OLT to date is arthroscopic bone marrow stimulation (BMS)^6,7^. The advantages of arthroscopic BMS over other treatments are its relative minimal invasiveness, low cost, technical feasibility, and wider availability in less-resourced health-care systems^7,8^. Moreover, arthroscopic BMS has shown good and reliable results up to mid-term follow-up^9,10^. There is a concern in the literature, however, that clinical results may deteriorate and/or ankle osteoarthritis may progress over time because biomechanically inferior fibrocartilage is formed after BMS^10-14^. This may result in recurrent symptoms and the need for subsequent revision surgery^15^.

The current literature on long-term outcomes following BMS for OLT can be considered limited^11^. As such, the long-term sustainability of BMS for OLT, with a specific focus on survival outcomes, is understudied and there is sparse evidence on baseline patient and lesion factors that may influence long-term survival free from revision^11^. The primary aim of the present study was therefore to assess the 10-year survival following arthroscopic BMS for OLT. The secondary aims were to evaluate the median time to revision and the influence of baseline patient and lesion characteristics on survival. These outcomes are of critical importance for patients and physicians during patient counseling and shared decision-making, and could aid in optimizing patient outcomes^7,16^.

## Materials and Methods

This was a single-center retrospective cohort study. Our institution is an academic tertiary referral hospital that specializes and is (inter)nationally accredited in the treatment of ankle cartilage injuries. This study was approved by the institutional review board (MEC 08/326) and performed in accordance with the Declaration of Helsinki.

### Patient Selection

All patients who underwent arthroscopic BMS for a symptomatic OLT and had a minimum of 10 years of follow-up (i.e., were treated before January 2013) were eligible for inclusion. BMS was defined as arthroscopic debridement with or without microfracture. Patients who underwent treatment had a symptomatic OLT with pain and/or associated clinical symptoms (swelling, locking, etc.) and failure of initial nonoperative management^8^. BMS was performed in accordance with previously published techniques^17,18^. Patients were identified, according to the inclusion and exclusion criteria, from an existing historical database of patients with a computed tomography (CT)-confirmed OLT^19^. After patients were identified, they were contacted by phone for their participation in the study. If patients could not be reached by phone, 2 subsequent emails and/or letters were sent. If no response was received or if the patient had died, they were considered lost to follow-up (i.e., nonresponders). The exclusion criteria are outlined in Table I.

**TABLE I:** Exclusion Criteria

Revision surgery due to postoperative infection related to the index procedure, requiring surgical debridement of the ankle
No preoperative CT scan or MRI available
Coexisting osteochondral lesion of the tibial plafond (OLTP) on preoperative CT or MRI
Preoperative advanced tibiotalar joint osteoarthritis, defined as severe joint-space narrowing or Kellgren-Lawrence grade ≥3
Objection to study participation

### Outcome Measures

The primary outcome of this study was the 10-year cumulative survival rate, defined as the proportion of ankles that had not undergone revision surgery at 10 years after the index procedure. The index procedure in patients with multiple arthroscopic BMS procedures was defined as the procedure that had the longest follow-up at our institution. Revision surgery was defined in accordance with the definition established by the International Consensus Meeting on Cartilage Repair of the Ankle^15^. More specifically, the present study defined revision surgery as any surgical procedure for a recurrent OLT after the index procedure, according to the OLT treatment categories defined by Dahmen et al.^6^, or tibiotalar joint arthrodesis, total ankle replacement, amputation, or ankle realignment surgery.

The secondary outcomes of this study were the time to revision, 15 and 20-year revision rates, and associations of predictive baseline patient and lesion factors with revision surgery. According to the statistical principles of survival analysis, a 10:1 ratio of the number of failures to the number of predictive baseline factors was considered acceptable^20^. Therefore, before the start of the study, a hierarchy of possible dichotomous predictive factors was defined according to the current evidence-based literature^21-28^ in order to determine which factors were to be analyzed. The following hierarchy was established: (1) lesion size (≤100 versus >100 mm^2^), (2) a primary versus non-primary lesion (i.e., following failed primary surgery), (3) the presence versus absence of subchondral cysts on preoperative imaging, (4) a body mass index (BMI) of ≥30 versus <30 kg/m^2^, and (5) sex.

### Data Collection

Baseline patient, lesion, and treatment characteristics were collected from the patient electronic health records. Patient characteristics included sex, age at the time of surgery, BMI, laterality, etiology (traumatic or non-traumatic), the presence of ankle instability (defined as patient-reported recurrent spraining and/or subjective ankle instability, laxity during physical examination, or as concluded by the physician in the clinical report^19^), and previous ankle surgeries. Lesion characteristics included primary or non-primary lesion type as well as radiographic characteristics. Treatment characteristics included follow-up time (in years), lesion debridement with or without microfracture, anterior or posterior arthroscopy, and any concomitant procedures.

Outcome measures were collected from the patient electronic health records as well as by phone interview to confirm whether patients had or had not undergone revision surgery. If a revision surgery had been performed, the following data were collected: type of revision surgery (according to the previously described categorization of surgical procedures for the OLT), revision surgery date, and reason for revision surgery.

### Radiographic Evaluation

Preoperative lesion characteristics were collected by 2 independent raters (Q.G.H.R. and M.B.A.) on CT scans (n= 254). If no preoperative CT scan was available, magnetic resonance imaging (MRI) was utilized (n = 8). The following lesion characteristics were collected: lesion size (anterior-posterior and medial-lateral directions as well as depth) measured in millimeters and converted to the area as described by Choi et al.^29^ and to the volume as described by Angthong et al.^30^, dominant lesion morphology as described by Rikken et al.^19^, the presence of cysts, and the location according to a 9-gird scheme^31^. A consensus meeting was held in case of disagreement on the lesion characteristics between the 2 raters, and if no agreement could be achieved, a third rater (J.D.) made the decision.

### Statistical Analysis

Data analyses were conducted using Stata (version 15; StataCorp). A 2-sided p value of <0.05 was considered significant. Baseline dichotomous and categorical data are reported as counts with percentages, and continuous data are reported as means with standard deviations. Data were visually assessed for normality using histograms. The primary outcome, namely the cumulative 10-year survival rate, was analyzed by means of the Kaplan-Meier survival method and reported with the 95% confidence interval (CI). Patients who were lost to follow-up were censored for the primary outcome at the time of their latest follow-up visit. No power analysis was performed for the primary outcome, as this study involved an observational outcome in a single cohort. The median time to revision surgery was calculated along with the interquartile range (IQR). The effects of baseline predictive factors on survival were determined using univariate Cox regression analysis and reported as hazard ratios (HRs) with 95% CIs. All variables that were significantly associated with revision in the univariate analyses (at an adjusted significance level of 0.1) were entered into a multivariable Cox regression model. Backward selection was used to identify the factors that remained predictive of revision (at a significance level of 0.05). Multiple imputation was performed for missing data (BMI for 74 ankles [28%] and lesion size for 1 ankle [0.4%]), under the assumption that data were missing at random. All model variables and 2 auxiliary variables (age, sex) were used for imputation of the missing data. Five data sets were created, and pooling of the outcome was performed according to the Rubin rules^32^. A sensitivity analysis was also performed by means of complete-case analysis. A description of further subanalyses as well as interrater and intrarater reliability measurements are provided in Appendix Supplementary Materials 1.

## Results

**Fig. 1:**
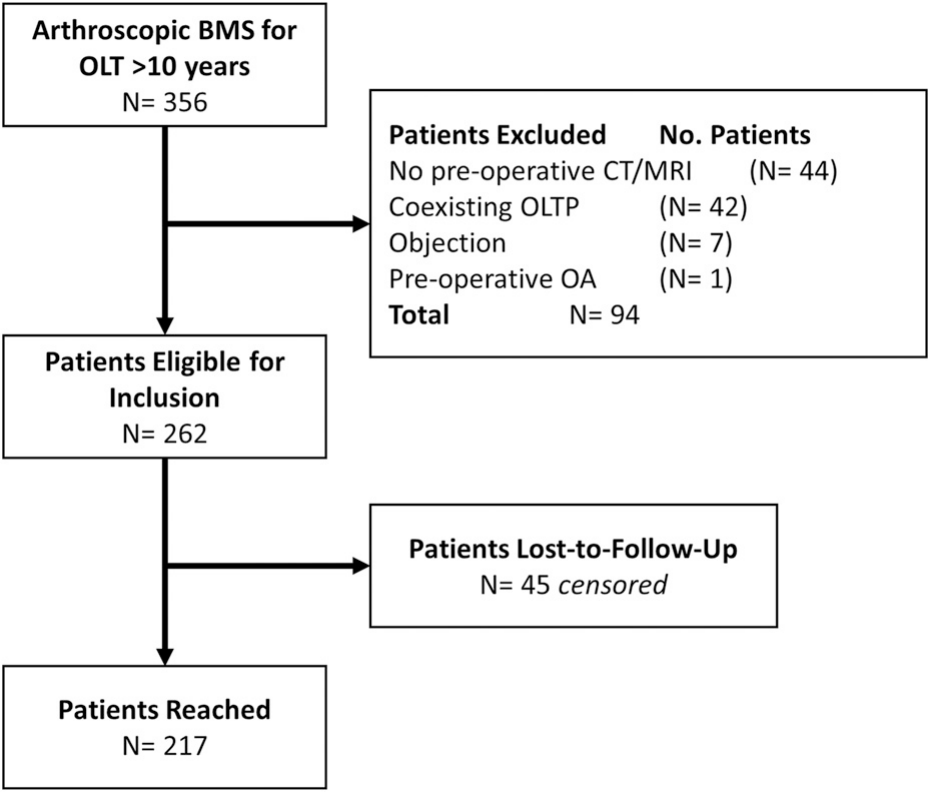
Flowchart of patient selection. OLTP = osteochondral lesion of the tibial plafond, and OA = osteoarthritis.

At the time of final follow-up, at a mean of 15.3 ± 4.8 years postoperatively, 262 cases were eligible for inclusion; the patient was reached in 217, and the other 45 were censored at a mean of 1.8 ± 3.4 years (Fig. 1). One patient had a bilaterally treated OLT (i.e., 2 cases). An overview of the baseline patient, treatment, and lesion characteristics is shown in Table II. There were no significant differences in baseline characteristics between the patients who were reached and those who were censored, except for a longer follow-up time for censored patients (i.e., nonresponders). The outcomes of the interrater and intrarater reliability measurements are shown in Appendix Supplementary Materials 2.

**TABLE II:** Baseline Patient, Treatment, and Lesion Characteristics*

	All Cases (N = 262)		Responders (N = 217)		Nonresponders (N = 45)		P Value
	Value	% with Data	Value	% with Data	Value	% with Data	
Sex: male	162 (62%)	100	130 (60%)	100	32 (71%)	100	0.2
Age *(yr)*	32.3 ± 11.7	100	32.1 ± 11.6	100	33.0 ± 12.2	100	0.8
Time to event† *(yr)*	10.9 ± 7.3	100	12.8 ± 6.4	100	1.8 ± 3.4	100	NA
Follow-up time‡ *(yr)*	15.3 ± 4.8	100	14.9 ± 4.6	100	17.1 ± 5.3	47	**<0.01**
BMI *(kg/m*^*2*^*)*	25.7 ± 4.4	72	25.5 ± 4.2	77	26.8 ± 5.5	100	0.2
Laterality: right	137 (52%)§	100	113 (52%)	100	24 (53%)	100	0.9
Lesion etiology		93		93		93	0.8
Non-traumatic	64 (26%)		53 (26%)		11 (26%)		
Traumatic	180 (74%)		149 (74%)		31 (74%)		0.1
Ankle instability	50 (20%)	93	40 (20%)	94	10 (25%)	89	
Treatment characteristics							
Arthroscopic approach		97		97		98	0.8
Anterior	197 (77%)		162 (77%)		35 (80%)		
Posterior	58 (23%)		49 (23%)		9 (20%)		
Microfracture		99		99		98	0.5
Debridement only	35 (13%)		31 (14%)		4 (9%)		
Debridement with microfracture	225 (87%)		185 (86%)		40 (91%)		
Concomitant surgery#		100		100		100	0.1
No. of ankles	107 (41%)		84 (39%)		23 (51%)		
Total no. of procedures	129		99		30		
Resection of osseous impingement	55(43%)		41 (41%)		14 (47%)		
Resection of soft-tissue impingement	13 (10%)		11 (11%)		2 (7%)		
Removal of loose body	39 (30%)		32 (32%)		7 (23%)		
FHL release	11 (9%)		7 (7%)		4 (13%)		
Resection of os trigonum	8 (6%)		6 (6%)		2 (7%)		
ATFL/capsular shrinkage	3 (2%)		2 (2%)		1 (3%)		
Lesion characteristics							
Primary lesion	212 (81%)	100	173 (80%)	100	39 (87%)	100	0.4
Presence of cyst	139 (53%)	100	113 (52%)	100	26 (58%)	100	0.5
Lesion morphology		100		100		100	0.6
Cyst	127 (48%)		102 (47%)		25 (56%)		
Crater	85 (32%)		73 (34%)		12 (27%)		
Fragment	50 (19%)		42 (19%)		8 (18%)		
Lesion location		100		100		100	0.4
Zone 1	19 (7%)		16 (7%)		3 (7%)		
Zone 2	1 (0.4%)		1 (0.4%)		0 (0%)		
Zone 3	10 (4%)		7 (3%)		3 (7%)		
Zone 4	87 (33%)		75 (35%)		12 (27%)		
Zone 5	4 (2%)		3 (1%)		1 (2%)		
Zone 6	29 (11%)		23 (11%)		6 (13%)		
Zone 7	63 (24%)		52 (24%)		11 (24%)		
Zone 8	6 (2%)		3 (1%)		3 (7%)		
Zone 9	43 (16%)		37 (17%)		6 (13%)		
Lesion size *(mm)*							
Anterior-posterior	10.7 ± 4.0	99	10.8 ± 4.0	99	10.4 ± 4.6	100	0.5
Medial-lateral	7.8 ± 3.3	100	7.8 ± 3.2	100	7.7 ± 3.4	100	0.5
Depth	6.5 ± 2.9	100	6.5 ± 3.0	100	6.2 ± 2.5	100	0.5
Lesion area *(mm*^*2*^*)*	73.1 ± 49.8	99	73.4 ± 49.8	99	71.8 ± 64.8	100	0.4
Lesion volume *(mm*^*3*^*)*	367.8 ± 385.5	99	369.4 ± 357.9	99	360.0 ± 502.1	100	0.4

* The values are given as the mean ± standard deviation or as the count with the percentage in parentheses. All percentages are of the number of cases with available data for the variable except for the individual concomitant procedures, which are of the total number of concomitant procedures. NA = not applicable, FHL = flexor hallucis longus, ATFL = anterior tibiofibular ligament.

† The time to event is the time from the index surgery to censoring (i.e., including the observation time with or without loss to follow-up) or to revision surgery.

‡ The follow-up time is from the index procedure to the time of inclusion.

§ 1 case had bilaterally treated OLT.

# This is the total number of procedures; a patient may have had >1 concomitant procedure.

### Survival Analysis

**Fig. 2:**
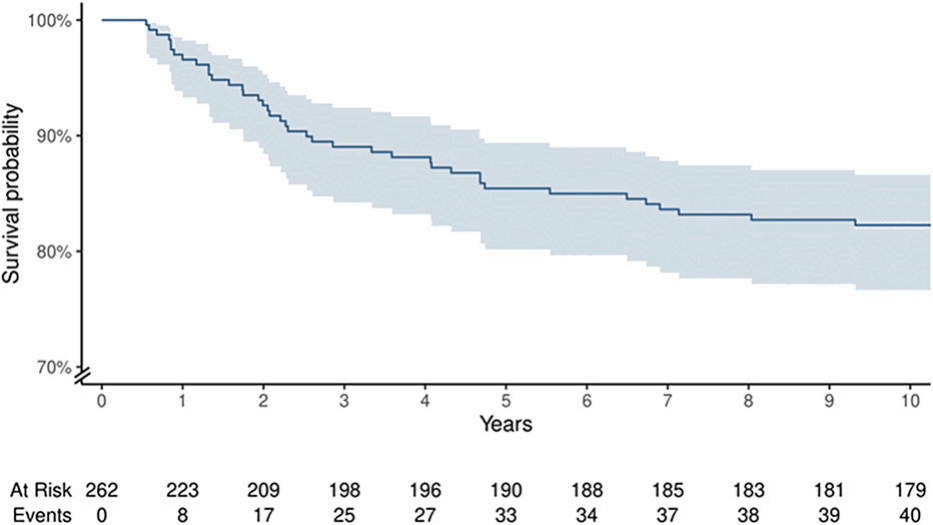
Kaplan-Meier survival curve at up to 10 years of follow-up. The survival rate is shown using a y axis from 70% to 100%; shading represents the 95% CI. The number of patients at risk and the cumulative number of events at each given time are listed below.

The 10-year cumulative survival rate of arthroscopic BMS in the 262 cases available in this cohort was 82.3% (95% CI: 76.6% to 86.7%). The Kaplan-Meier survival curve is shown in Figure 2. The median time to revision was 2.4 years (IQR: 1.3 to 5.1 years). A subanalysis revealed no significant differences in baseline patient and lesion characteristics between patients who underwent revision early (<2.5 years of follow-up) or late (>2.5 years of follow-up) revision (see Appendix Supplementary Materials 3).

At 15 and 20 years of follow-up, the cumulative survival rate was 81.6% (95% CI: 75.8% to 86.1%, n = 62) and 77.7% (95% CI: 68.5% to 84.0%, n = 33), respectively.

### Baseline Factors and Survival Outcomes

As 44 events occurred in the study population, 4 of the 5 predetermined baseline factors (selected according to the prospectively established hierarchy) were analyzed for their association with survival. Table III shows the hazard ratio for each variable. The Kaplan-Meier curves for all variables are shown in Appendix Supplementary Materials 4.

**TABLE III:** Cox Regression Analysis of Baseline Factors Associated with Failure*

Analysis and Variable	HR (95% CI)	P Value
Univariate		
Lesion size		
≤100 mm^2^	Reference	
>100 mm^2^	0.93 (0.47-1.83)	0.82
Lesion type		
Primary	Reference	
Non-primary	1.78 (0.93-3.41)	**0.08**
Presence of cyst		
No	Reference	
Yes	1.02 (0.57-1.85)	0.94
BMI		
<30 kg/m^2^	Reference	
≥30 kg/m^2^	3.04 (1.44-6.43)	**<0.01**
Sex†		
Male	Reference	
Female	0.60 (0.33-1.08)	0.09*
Multivariable		
Lesion type		
Primary	Reference	
Non-primary	1.57 (0.83-3.03)	0.18
BMI		
<30 kg/m^2^	Reference	
≥30 kg/m^2^	2.82 (1.30-6.1)	**0.01**
Final model		
BMI		
<30 kg/m^2^	Reference	
≥30 kg/m^2^	3.04 (1.44-6.43)	**<0.01**

* After multiple imputation. HR = hazard ratio, CI = confidence interval.

† Sex was not included in the formal analysis because of underpowering, as described in the Materials and Methods section. However, it is shown here to support the secondary analysis and should be interpreted as such.

**Fig. 3:**
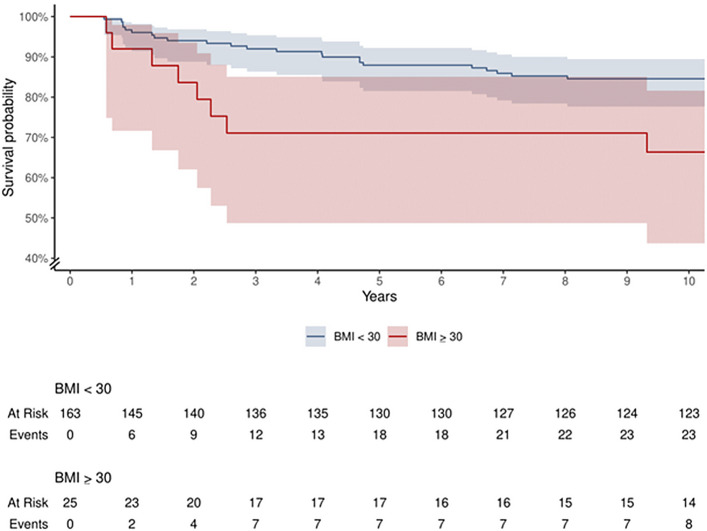
Kaplan-Meier survival curve comparing the survival of patients with and without obesity. The survival rate is shown using a y axis from 40% to 100%; shading represents the 95% CI. The number of patients at risk and the cumulative number of events at each given time are listed below for each group.

A baseline BMI of ≥30 kg/m^2^ was significantly associated with a higher likelihood of revision following BMS (HR: 3.0 [95% CI: 1.44 to 6.43], p < 0.01) (Fig. 3.) A comparison of baseline characteristics is shown in Appendix Supplementary Materials 5.

The complete-case analysis, which is shown in Appendix Supplementary Materials 6, found results comparable to those of the primary analysis.

## Discussion

The most important finding of this study is that 82% of ankles that underwent arthroscopic BMS procedures for an OLT remained free from revision at 10 years of follow-up. At 15 and 20 years of follow-up, the cumulative survival rate was 82% and 78%, respectively. Furthermore, this study found that obesity (BMI ≥ 30 kg/m^2^) may be associated with a higher likelihood of revision surgery.

Survival in orthopaedics is a dynamic outcome that incorporates functional outcomes, pain, complications, return to sports and work, and mental aspects, as well as a patient’s experience and expectations^16,33,34^. Outcomes in 1 or more of these domains must reasonably be below the level of satisfaction in order for a patient to consider revision surgery. In contrast to arthroplasty studies, few studies to date have specifically focused on survival outcomes in joint preservation surgery for osteochondral lesions of the ankle^22,35,36^. This study sought to evaluate the long-term clinical survival after arthroscopic BMS for OLT and observed a high rate of survival that was sustained over time. When comparing the outcomes of this study with the literature, it should be noted that a limited number of studies on the long-term outcomes following BMS for OLT have been published, with even fewer assessing its survival^11,13,22,37-40^. A recent systematic review found an overall survival rate of 93% in 317 ankles from 5 studies with long-term outcomes^11^. The study by Park et al.^22^ was the largest contributor, with 202 of the 317 ankles. That study found a survival rate of 94% at a mean of 14 years of follow-up and good clinical outcomes. The second largest study, by van Bergen et al.^13^, included 50 patients with primary OLT at a mean follow-up of 10 years; it found a survival rate of 90% and excellent to good Berndt and Harty scores in 78%. It can be hypothesized that the 10% higher survival rate reported in the literature compared with the present study is due to patient selection^11^. First, the aforementioned systematic review included almost exclusively (96%) primary lesions^11^, whereas the present study includes 19% non-primary lesions. Even though the association of a non-primary lesion type with decreased survival did not reach significance, the inclusion of the relatively high proportion of non-primary lesions could have impacted the survival rate, as there is evidence that these lesions may result in inferior patient-reported outcome measures (PROMs) compared with primary lesions^24^. Second, the present study was performed in a cohort of patients from a tertiary referral hospital recognized as an (inter)national expert center in the diagnosis and treatment of OLT. Thus, the patients may have been inherently more challenging to treat because of the presence of more risk factors or higher patient expectations. However, it should be noted that an 82% to 94% survival rate at 10 years is to be expected following arthroscopic BMS, according to the literature and the findings of this study^11,13,22,37-40^. Moreover, this study also calculated survival rates at 15 and 20 years of follow-up, and survival appeared nearly stable beyond 10 years, which is an encouraging finding for the practice of BMS for OLT.

It has been hypothesized that BMS fails over time due to the progression of osteoarthritis, and 28% of cases reported in the literature showed radiographic progression of degenerative changes; however, only 4% of cases were reported to have actual joint-space narrowing^11^. Radiographic follow-up was not included in the present study, as our treatment protocol does not include long-term radiographic evaluation of patients who are doing well clinically. Thus, we believe that including the available radiographic studies of the patients who returned for re-evaluation of their ankle would not have been representative of the cohort as a whole, may have overestimated the progression of osteoarthritis due to selection bias, and could therefore have resulted in incorrect conclusions. The revisions in this cohort included only 5 procedures (2% of all cases) that consisted of tibiotalar arthrodesis or arthroplasty, suggesting that the rate of symptomatic end-stage osteoarthritis was low or that its onset would present outside the time window of this study.

### Baseline Factors Associated with Survival

Our findings suggest that patients with obesity (BMI ≥ 30 kg/m^2^) have a decreased survival rate. The effect of BMI on clinical outcomes remains debated. A recent study by Koh et al.^27^ reported that a BMI of ≥25 kg/m^2^ was not associated with decreased outcomes at 2 years of follow-up in 252 patients who underwent arthroscopic BMS. Indeed, the aforementioned authors found that a higher BMI appeared to be weakly associated with improved outcomes. In contrast, several studies found that BMI was associated with poorer PROMs^41,42^ and decreased survival^22^. Based on these reports and the findings of the present study, advising weight reduction can be recommended as best practice when counseling obese patients regarding surgery. It could be hypothesized that an association between obesity and poorer survival outcomes is present because the elevated BMI exaggerates the biomechanical stress on fibrocartilage or surrounding hyaline cartilage and subchondral bone in the highly congruent tibiotalar joint, leading to treatment failure. On the other hand, we hypothesize that obesity may also represent a variable that encompasses a multifactorial risk profile, such as lower social economic status, general health, or mental health, which could affect PROMs^43,44^. Additionally, we note that it is important to consider the statistical frailty due to the relatively small number of patients with obesity in this study, which precludes definitive conclusions regarding the relationship between BMI and survival.

Among the other baseline factors assessed, the associations of long-term survival with the lesion size, a non-primary lesion type, the presence of preoperative cysts, and patient sex did not reach the level of significance. These findings are not in line with previous studies finding a relationship between these baseline characteristics and PROMs^21-28^, which may be because the outcome of survival free from revision incorporates not only PROMs but also mental health, physical functioning, work and sport activities, etc. However, we did observe a nonsignificant trend toward poorer survival for non-primary OLT and for female patients (see Appendix Supplementary Materials 5). These trends could show a significant association in larger samples, which would be highly clinically relevant, and both factors should therefore be investigated further.

### Limitations and Strengths

The results of this study should be interpreted in the context of its design, and it is not without limitations. First, it was performed retrospectively using a single-center cohort. Additionally, it did not include clinical and radiographic follow-up data, but instead focused on revision outcomes. In our institution, a recurrent OLT is treated according to shared decision-making using a stepwise approach before revision surgery is chosen. As such, recurrent lesions are first treated with a period of nonoperative treatment and any concomitant pathology is addressed. Second, 17% of patients could not be contacted; however, that is to be expected in long-term follow-up studies and is in line with prior studies^22^. Furthermore, no differences in baseline characteristics were observed between responders and nonresponders. Third, statistical frailty and overfitting of the models for assessing the effects of baseline characteristics on survival may be present, given the small number of patients per group. However, we sought to limit this effect by prospectively establishing a hierarchy for assessing these variables.

The strengths of this study are its focus on survival outcomes in what we believe to be the largest cohort of patients with long-term follow-up after BMS for OLT to date and its predetermined statistical plan for the evaluation of prognostic baseline factors. Moreover, the data extraction and radiographic measurements were performed by 2 authors and their results showed excellent interrater and intrarater reliability. We recommend future studies to substantiate our findings regarding the survival rate and risk factors for failure as well as osteoarthritis by utilizing large (international) multicenter cohorts of patients with long-term follow-up following BMS for OLT.

### Conclusions

At a minimum follow-up of 10 years, the survival rate free from revision was 82% after arthroscopic BMS for OLT. At 15 and 20 years of follow-up, survival appeared to remain stable. Obesity (BMI ≥ 30 kg/m^2^) was associated with a higher likelihood of revision surgery. This risk factor should be incorporated into the treatment algorithm for OLT when counseling patients regarding surgery.

## Appendix

Supporting material provided by the authors is posted with the online version of this article as a data supplement at jbjs.org (http://links.lww.com/JBJS/I10).
